# Data on hyoid bone position in class I and class III malocclusions

**DOI:** 10.6026/973206300220859

**Published:** 2026-02-28

**Authors:** Yohan Verghese, Arvind Kumar, Soghyia Talib Abrahim Al Hamar, Jaikrishnan Ramachandran, Roni Bose

**Affiliations:** 1 Department of Orthodontics and Dentofacial Orthopedics, Saveetha Dental College, Chennai, Tamilnadu, India; 2 Department of Orthodontics and Dentofacial Orthopedics, MOHAP, UAE; 3 Specialist Orthopedic Surgeon, Al Qassim Hospital, Saudi Arabia; 4 Smile Design Dental Clinic, Kerala, India

**Keywords:** Class I malocclusion, Class III malocclusion, hyoid bone, hyoid bone position

## Abstract

In Class I and III malocclusion the position of the hyoid varies. We can able to predict the position of the hyoid when comparing its position between Class I malocclusion and Class III malocclusion. In this meta-analysis search was done using various keywords like "hyoid bone", "Class I", "Class III", "hyoid cephalometrics", "Pharyngeal airway", Pharyngeal changes", "Uvulo-glossopharyngeal" and "hyoid triangle". It was found that there was a definite correlation between the hyoid bone position in Class I and Class III malocclusion. It was found that the hyoid was more superiorly and anteriorly positioned in a class III malocclusion when compared to a class I malocclusion.

## Background:

Edward Angle released his "Classification of Malocclusion" in 1899, one hundred years ago. According to Angle, the mesiobuccal cusp of the upper first permanent molar occludes with the mesiobuccal groove of the lower first permanent tooth in a Class I molar relation. The lower first permanent molar is mesial to the upper first permanent molar by a premolar width or a cuspal width in a Class III molar relation, and the distobuccal cusp of the upper first permanent molar occludes with the mesiobuccal groove of the lower first permanent molar in a Class II molar relation [1]. The mandible, pharynx, and cranium's muscles, ligaments, and connective tissues suspend the hyoid bone, which is remarkable in that it lacks bony articulation. Thus, the joint action of the suprahyoid and infrahyoid muscles, as well as different oral activities closely related to tongue activity, determines the position of the hyoid bone. According to available data, assessing the hyoid bone position may provide a more precise measurement of mandibular position and its impact on the airway [2]. There is a clear correlation between hyoid bone location and malocclusion. Therefore, it is of interest to understand that the hyoid is most superiorly and anteriorly placed in a class III when compared to a class I malocclusion. The Class I malocclusion views the hyoid bone as being positioned between the Class II and III malocclusions.

## Materials and Methods:

The study was done based on cephalometric finding of previous studies. They are patients who had both types of Angles malocclusions. They were selected from studies done in different parts of the world namely Estonia and Greece in the West, to the Iranians in the Middle East, to the Koreans and Indians in the East. Comparisons - All patients had the same cephalometric measurements taken and they were compared with each other in different populations in the world to show the outcomes.

## Outcomes:

The patients' values would show that the position of the hyoid in an Angles class III malocclusion would be superior and anterior to an Angle's class I malocclusion.

## Protocol registration:

This review followed the preferred reporting items for systematic reviews and Meta-analysis. The Review Manager 5.3 software was used to analyse the data.

## Search strategy:

Using search databases of Pubmed, Elsevier, Scopus and Google, the terms Hyoid bone, Malocclusion and Position were used to perceive any correlations between the position of the hyoid and malocclusion. The purpose was to ascertain whether the hyoid bone would have a particular position depending on the malocclusion of the patient. The search terms were selected based on the requirement that the position of the hyoid would have an effect on malocclusion at the end of orthodontic treatment and on it stability thereafter. The search terms were "hyoid bone", "ClassI", "Class III", "hyoid cephalometrics", "Pharyngeal airway", "Hyoid movement", "Hyoid changes", "Pharyngeal changes", "Uvulo-glossopharyngeal" and "hyoid triangle".

## Selection of studies:

## Inclusion and exclusion criteria:

The inclusion criteria for these studies mainly centered on the fact that they used the same angles for measurement of the hyoid bone position. The exclusion criteria were automatically any articles which mentioned the hyoid bone but did not use the same points for the measurement of the hyoid bone and its position with regard to the various cephalometric measurements made in the seven primary articles. The seven primary articles used in this review having similar points and angles used for measurements had all lateral cephlograms taken in occlusion and traced. Mortazavi *et al.* article based on Iranian population used the ANB angle to classify the patients into different skeletal patterns [3]. The ANB angle was also used as the reference for classification by Deshkar *et al.* article based on the Estonian population [4]. The Adamidis *et al.* article based on the Greek population used dental classifications of malocclusions with esthetically pleasing facial structure compared with prognathic mandibles to classify the patients as did the Song *et al.* article on the Korean population [5, 6]. The Adamidis *et al.* article on the Greek population, the Mortazavi article on the Iranian population, the Song *et al.* article on the Korean population and the Peets article on the Estonian population did separate them in to genders [4, 5-6]. In Mortazavi study on the Iranian population the minimum age of 18 years was mentioned to take part in the study [3]. The Adamidis study on the Greek population mentioned an age group of 10 to 13 years [5]. Deshkar *et al.* study on the Estonian population mentioned an age group of 10 to 13 years with an average age of 12.1 for the boys and 11.4 for the girls [4]. The Song *et al.* article on the Korean population mentioned a 6.6 to 11.7 year age group with a mean of 9.1 years [6]. The Song et al article on the Korean population, the Adamidis article on the Greek population, Deshkar *et al.* article on the Estonian population did not mention growth pattern as exclusion criteria [4, 5, 6, 7-8]. The Mortazavi article on the Iranian population mentioned excluding severe vertical and horizontal growth pattern individuals [3]. The position of the hyoid was measured using the following linear and angular measurements:- APH-At, APH-S, APH-Pog on S, APH-Pog, APH-A, APH-A on S, APH-N, APH-FH, PPH-FH, GoP-AFH, LAH-FH, LAH-PBR, LAH-MP, LAH-BaN, LAH-PP, H-MP, C3HY, HRGN, Hyoid angle. All studies that had these angular and linear measurements with patients who were in adolescence or adults were selected. The statistical data was analysed using a Statistical Package for Social Science software and the meta-analysis using the review manager 5.3 software.

## Results:

There are clear cut studies which were published by Adamidis & Spyropoulos in 1992, Song & Kim in 1999 and Deshkar and Jagomagi in 2006 to show the difference of hyoid position in class I, II and III malocclusion [4, 5-6]. The advantage of these studies was the fact that they used exactly the same points and measurements thereby allowing us to see if there was a differential in the values between class I and III malocclusions. The population used was different as the 1999 study was done in Korea rather than Greece and the third study in Estonia. However, as the selection criteria was the various accepted types of malocclusions e.g. class I and III, it would not make a difference in viewing whether the position of the hyoid moved posteriorly in class I when compared to the class III. As you can see the hyoid is more anteriorly placed in class III malocclusion than in class I and also the inclination of the hyoid is more horizontal than vertical in class III than class I. In looking closely we can see that the hyoid has moved anteriorly when it is a class III malocclusion, in all 3 populations when compared with the class I malocclusion. Furthermore, the inclination of the hyoid in more horizontal in the class III malocclusion when compared with class I malocclusions in all 3 populations. The only exception to the inclination was the value LAH-BaN value between class I and III in the Estonian population [5]. Therefore from 6 different values only 1 value showed an aberration which brings a percentage of error to 16.7%.In APH-AT values showing that, the distance is greater in class III when compared to class I and least in class II when compared to class I which reinforces our ideal on the position of the hyoid. Although it is expected in APH-Pog on S and direct should show an increased value in class II when compared to I and decreased in class III when compared to I, we can see decrease in class II when compared to class I in the Korean population but the class III to class I prediction matches perfectly [9].

In the same measurement in the Estonian population we can see that between the class III and class I values the prediction is that there should be decreased but in shows an increase while in the class II to class I the values match the prediction perfectly [5]. In the measurement of PPH-FH the expectation is that the class II value will be decreased when compared to the class I value. In the Estonian population this does not hold true as the class II value is higher than the class I however the class III value matches the prediction perfectly [5]. The APH-FH value should show a decrease in the class III value when compared to the class I. The Estonian population values do not ascribe to this and shows an increase in the class III when compared to the class I value; however the class II values match the prediction perfectly [5]. The GOP-APH value should show a decrease in the class III value when compared to the class I but the Estonian population shows and increase in the class III value when compared to the class I. The class II value matches the prediction as expected [5]. The LAH-PBR angle should show an increase in the class II values and a decrease in the class III values when compared to the class I values but shows a decrease in the class II and an increase in the class III values when compared to the class I. This aberration is seen in the Estonian population alone [5]. As can be seen the LAH-MP value should be increased in class I and decreased in class III according to the prediction. Class III to class I values are in sync with what is anticipated. If can be seen however the expected increase in a class II is seen in the Indian Population [4]. The inclination of LAH PP decreases in class III malocclusion when compared to class I malocclusion which clearly illustrates the hypotheses that the hyoid is more posteriorly located in class I than class III malocclusion. In the Iranian population the distance of C3Hy should have been greater in class III than I but it is not so in this study [3]. However, in the Korean population it supports the hypothesis that the distance should be greater in class III than in class I [9]. In terms of HRGn all the values oppose the hypothesis that the values should be greatest in class III malocclusions followed by class I malocclusions. The results in the table above supports the hypothesis that the hyoid is more anteriorly inclined in all populations in the class III than the class I the only exception to this being in the Iranian population where the inclination of the class III shows a more posterior inclination.

## Meta-analysis:

The pooled Fixed effect meta-analysis for ApH-At in Class I and Class III malocclusion population shows the mean difference of -0.70 (-2.48,-1.09) mm. Further, ApH-At in Class III malocclusion the distance is more compared to Class I malocclusion for the population (p=0.44) showing a more anterior position of the hyoid in the class III. The pooled Fixed effect meta-analysis for ApH-S in Class I and Class III malocclusion population shows the mean difference of -6 (-8.51,-3.48) mm. Further, ApH-S in Class III malocclusion the distance is more compared to Class I malocclusion for the population (p=0.00001) showing a more anterior position of the hyoid in class III. The pooled Fixed effect meta-analysis for ApH-Pog on S in Class I and Class III malocclusion population shows the mean difference of -1.38 (-0.24-3) mm. Further, ApH-Pog on S in Class III malocclusion the distance is less compared to Class I malocclusion for the population (p=0.10) showing a more anterior position of the hyoid in class III. The pooled Fixed effect meta-analysis for ApH-Pog in Class I and Class III malocclusion population shows the mean difference of 1.13 (-0.51-2.78) mm. Further, ApH-Pog in Class III malocclusion the distance is less compared to Class I malocclusion for the population (p=0.18) showing a more anterior position of the hyoid in class III. The pooled fixed effect meta-analysis for ApH-A on S in Class I and Class III malocclusion population shows the mean difference of 5.87 (3.90-7.85) mm. Further, ApH-A on S in Class III malocclusion the distance is less compared to Class I malocclusion for the population (p=0.00001) showing a more anterior position of the hyoid in class III. The pooled fixed effect meta-analysis for ApH-N in Class I and Class III malocclusion population shows the mean difference of 9.517 (7.30-11.73) mm. Further, ApH-N in Class III malocclusion the distance is less compared to Class I malocclusion for the population (p=0.000001) showing a more anterior position of the hyoid in the class III as anticipated in the population. The pooled Fixed effect meta-analysis for PPH-FH in Class I and Class III malocclusion population shows the mean difference of -2.07(-4.97-0.84) mm. Further, PPH-FH in Class III malocclusion the distance is more compared to Class I malocclusion for the population (p=0.16) showing a more inferior position of the hyoid in the class III as anticipated in the population. The pooled Fixed effect meta-analysis for APH-FH in Class I and Class III malocclusion population shows the mean difference of 1.26(-1.26-3.79) mm. Further, APH-FH in Class III malocclusion the distance is more compared to Class I malocclusion for the population (p=0.002) showing a more superior position of the hyoid in class III. The pooled Fixed effect meta-analysis for GoP-APH in Class I and Class III malocclusion population shows the mean difference of -1.40(-3.42-0.62) mm. Further, GoP-APH in Class III malocclusion the distance is more compared to Class I malocclusion for the population (p=0.17) showing a more superior position of the hyoid in class III. The pooled fixed effect meta-analysis for LaH-FH in Class I and Class III malocclusion population shows the mean difference of 5.87(3.90-7.85) degrees. Further, LaH-FH in Class III malocclusion the degree is more compared to Class I malocclusion for the population (p=0.00001) showing a more anterior and superior position of the hyoid in class III. The pooled Fixed effect meta-analysis for LaH-PBr in Class I and Class III malocclusion population shows the mean difference of -1.33(-4.13-1.46) degrees. Further, LaH-PBr in Class III malocclusion the degree is less compared to Class I malocclusion for the population (p=0.35) showing a more anterior and superior position of the hyoid in class III.

The pooled Fixed effect meta-analysis for LaH-MP in Class I and Class III malocclusion population shows the mean difference of -0.74 (-2.78-1.30) degrees. Further, LaH-MP in Class III malocclusion the degree is less compared to Class I malocclusion for the population (p=0.48) showing a more anterior and superior position of the hyoid in the class III. The pooled Fixed effect meta-analysis for LaH-BaN in Class I and Class III malocclusion population shows the mean difference of -0.18(-2.77-2.40) degrees. Further, LaH-BaN in Class III malocclusion the degree is less compared to Class I malocclusion for the population (p=0.89) showing a more anterior and superior position of the hyoid in class III. The pooled Fixed effect meta-analysis for LaH-PP in Class I and Class III malocclusion population shows the mean difference of 2.6 (0.35-4.85) degrees. Further, LaH-PP in Class III malocclusion the degree is less compared to Class I malocclusion for the population (p=0.02) showing a more anterior and superior position of the hyoid in the class III. The pooled fixed effect meta-analysis for APH-A in Class I and Class III malocclusion population shows the mean difference of 5.89 (4.06-7.71) mm. Further, APH-A in Class III malocclusion the distance is less compared to Class I malocclusion for the population (p=0.00001) showing a more anterior position of the hyoid in the class III as anticipated in the population. The pooled Fixed effect meta-analysis for H-MP in Class I and Class III malocclusion population shows the mean difference of 1.41 (0.25-2.57) mm. Further, H-MP in Class III malocclusion the distance is less compared to Class I malocclusion for the population (p=0.02) showing a more superior position of the hyoid in the class III. The pooled Fixed effect meta-analysis for C3-Hy in Class I and Class III malocclusion population shows the mean difference of 0.09 (-1.09-1.27) mm. Further, C3-Hy in Class III malocclusion the distance is more compared to Class I malocclusion for the population (p=0.88) showing a more anterior position of the hyoid in class III. The pooled fixed effect meta-analysis for H-RGn in Class I and Class III malocclusion population shows the mean difference of 3.63 (1.92-5.35) mm. Further, H-RGn in Class III malocclusion the distance is less compared to Class I malocclusion for the population (p=0.0001) showing a more anterior position of the hyoid in the class III as anticipated in the population. The pooled fixed effect meta-analysis for hyoid angle in Class I and Class III malocclusion population shows the mean difference of 3.63 (1.92-5.35) mm. Further, hyoid angle in Class III malocclusion the distance is less compared to Class I malocclusion for the population (p=0.0001) showing a more anterior and superior position of the hyoid in the class III as anticipated in the population.

## Discussion:

The first article of primary importance was done by Adamidis IP & Spyropoulos MN in 1992 which showed the clear relationship between the type of malocclusion in question and the hyoid bone. It was clearly seen from the readings mentioned that the hyoid in located more anteriorly in class III with less of an inclination when compared to the class I readings [5]. The second article of primary importance was by Song & Kim in 1999 [6] and they proved the work already laid down by Adamidis & Spyropulos [6]. The third article of primary importance was by Peets & Jagomagi, in 2006 where they confirmed most of the findings spoken of the in the previous articles with a few differences which can be ascribed to the population used [4]. The fourth study of significance was done in Iran by Mortazavi *et al.* and his findings also confirmed the relationship between the hyoid and the facial skeleton [3]. A pioneering work was the study done by Machado junior and Crespo. They did a study on the radiographic position of the hyoid bone in children with atypical deglutition. Atypical deglutition here is defined as the persistence of childlike deglutition after the replacement of deciduous dentition. They used millimetric measurements between hyoid and tuber as well as a second measurement between hyoid and mandibular plane. They found that there was a greater measurement found in individuals with atypical deglutition than with normal deglutition individuals [7]. In the study done by King, X ray relations between hyoid and cranial bones were constant before puberty [8]. In 1963, Frankel described the connection between mouth breathing and irregular position of the tongue, where the imbalance between suprahyoid and infrahyoid muscles leading to dorsal-caudal position of the hyoid bone [9]. In 1978, Graber found that after the treatment of subjects with chin cap therapy for three years the hyoid bone moved posteriorly and inferiorly relative to its initial position [10]. In 1983 a study done by Galvao, compared the position of the hyoid bone in subjects with different malocclusions and found that the position of the hyoid bone differs in various malocclusions [11]. In a longitudinal study done by Kolias in 1999, it was noted that the hyoid bone moves inferiorly with age and it is more pronounced in men. However; the anterior position remains stable [12]. In 2003 Kaduk *et al.* studied the position of the hyoid bone in children with clefts. The study showed that the position of the hyoid is more anterior and caudal in cleft children [13]. In 2005 Al haijja *et al.* found that there are differences in the position of the hyoid bone with respect to sagittal maxillomandibular relationships and that it is significantly correlated to the ANB angle [14]. Xiaolong *et al.* in 2017 found that the hyoid bone moved anteriorly and superiorly and rotated counterclockwise after treatment with preadjusted brackets followed with occlusal plane control [15]. Chung *et al.* in 2001 found that the hyoid bone position in patients altered by moving upward and forward when they were subjected to mandibular advancement and this did not reduce even 2 years after surgery [16].

A retrognathic mandible causes the hyoid bone to shift inferiorly and posteriorly, according to Khanna *et al.* Because the hyoglossus and middle constrictor muscles of the pharynx take the attachments from the greater horn of the hyoid, this displacement of the hyoid may impact the posture of the tongue in the oral cavity and the stretching of the pharyngeal wall [17]. According to Roy *et al.* (2017), the hyoid bone's position actively contributes to the balance of anterior and posterior muscle tension. It may also stretch the musculature after surgery, which may lead to relapse; for these reasons, it should be regularly assessed in pre-, post-treatment, and follow-up records [18]. When looking at the results most of the sources that were found supported the idea that the position of the hyoid changed at the end of orthodontic treatment. There was a definite variation in the position of the hyoid according to various sources. Khanna found that a retrognathic mandible leads to infero- posterior displacement of the hyoid bone and the author feels that this may be a more valid finding as the muscles of the hyoid are connected to the mandible [17]. Singh *et al.* stated that in Class II skeletal patterns, the hyoid bone is located at the back [19]. Awadalkreem *et al.* state that the positions of the hyoid bone and morphology of the soft palate differ across skeletal classes [20]. Gender dimorphism was observed by Acharya et al in both the dimensions of the pharyngeal airway and the position of the hyoid bone [21]. From the present meta-analysis we found that, hyoid is more anteriorly placed in class III malocclusion than in class I, the inclination of the hyoid is more horizontal than vertical in class III than class I and the hyoid is more posteriorly placed and vertically inclined in class I more than class III.

## Conclusion:

There is a difference in the position of the hyoid between class I and III malocclusions. There is also a definite lacuna about whether the class I or III malocclusion came to be due to a lack or excess of the mandible or maxilla which should also be accounted for in the study.
